# Have You Ever Seen the Impact of Crossing Fiber in DTI?: Demonstration of the Corticospinal Tract Pathway

**DOI:** 10.1371/journal.pone.0112045

**Published:** 2015-07-02

**Authors:** Dong-Hoon Lee, Ji Won Park, Sung-Hee Park, Cheolpyo Hong

**Affiliations:** 1 Department of Radiology, Johns Hopkins University School of Medicine, Baltimore, Maryland, United States of America; 2 Department of Physical Therapy, Catholic University of Daegu, Gyeongbuk-do, Republic of Korea; 3 Department of Physical Medicine and Rehabilitation, Chonbuk National University, Jeollabuk-do, Republic of Korea; 4 Center for Medical Metrology, Korea Research Institute of Standards and Science, Daejeon, Republic of Korea; Institute of Psychology, Chinese Academy of Sciences, CHINA

## Abstract

**Objective:**

The identification of the corticospinal tract (CST) pathway with a deterministic fiber tracking approach is limited because of crossing fibers, especially for the hand fibers of the CST due to the crossing superior longitudinal fasciculus (SLF). We examined a patient with congenital bilateral perisylvian syndrome (CBPS) who did not have the SLF, in order to visualize CST hand fibers that were not affected by crossing fibers.

**Methods:**

A 10-year-old girl without the SLF due to CBPS and three normal healthy subjects participated in this study. We used a deterministic fiber tracking algorithm, and the regions of interest (ROIs) were drawn in the posterior limb of internal capsule (PLIC) and the primary motor cortex. The apparent diffusion coefficient (ADC), fractional anisotropy (FA), relative anisotropy (RA), and volume ratio (VR) were measured based on the extracted fiber tracts.

**Results:**

The ADC values were not different between the normal subjects and the patient with CBPS. The FA, RA, and VR values of the normal subjects were similar, but were relatively higher than those of the patient with CBPS.

**Conclusion:**

Our results clearly show the impact of the crossing fiber for the hand motor fibers of the CST pathway with deterministic tracking algorithms in diffusion tensor tractography.

## Introduction

The corticospinal tract (CST), which is a major neural pathway between the motor cortex and the spinal cord, mediates voluntary movements [1,2]. The accurate identification of the CST pathway is important for clinical researchers and neuroscientists [3]. Diffusion tensor-magnetic resonance imaging (DT-MRI) fiber tractography has been widely used to identify neural fiber bundles.

However, the identification of the CST pathway with deterministic fiber tracking approaches is limited because of the effects of crossing fibers, especially the superior longitudinal fasciculus (SLF), which crosses over the hand fibers of the CST in the human brain. Crossed fiber tissue has been frequently observed in DT-MRI fiber tractography, and the tissue hampers accurate tracings of the hand fibers of the CST.

Congenital bilateral perisylvian syndrome (CBPS) is a type of cortical developmental abnormality involving poor operculation of the parietal lobe and a wide lateral sulcus [4–6]. Due to such abnormal development, CBPS naturally affects the formation of nerve fibers in the human brain.

In this study, we examined a patient with CBPS, who lacked the SLF, in order to visualize the hand fibers of the CST pathway, because they were not blocked by the crossing fibers of the SLF. To the best of our knowledge, there are no existing reports on the visualization of the hand fibers of the CST pathway with deterministic tracking algorithms that confirm the effects of the absence of the SLF in the human brain. Moreover, studies of three normal cases with similar ages were also performed in order to compare the DTI-derived parameters.

## Methods

### Participants

A 10-year-old girl who did not have a SLF due to CBPS participated in this study. Her gestational development was unremarkable, and her family history revealed no instance of consanguinity and no familial recurrence of CBPS. She had sucking difficulties in the perinatal period and infantile spasms during the first year of life, and developed partial seizures beginning at age 10. Moreover, she had mirror movement of the right hand during left-hand movement. Three right-handed normal healthy subjects (female: 3, mean age: 9.3 years) also participated in this study. They had no previous history of neurological or physical disorders.

The study and protocol were approved by the Chonbuk National University Hospital Institutional Review Board Committee and conducted according to the guidelines for research. All subjects understood the purpose of the study and provided written, informed consent prior to participation.

### Image Acquisition

Whole-brain DT-MRI was performed on a 1.5T Siemens Symphony MRI system with a single-shot echo planar imaging sequence with the following parameters: Echo time/Repetition time = 108/7,000 ms, Field of View = 200 mm^2^, matrix size = 128 × 128, Number of Excitations = 2, and a b-value of 1,000 s/mm^2^. We acquired 32 contiguous slices parallel to the anterior commissure-posterior commissure line with a 3.8-mm slice thickness with no gap in 12 different diffusion directions. The eddy current-induced image distortions were corrected by registering all diffusion-weighted images to nondiffusion-weighted (b = 0 s/mm^2^) images with 12-parameter affine multi-scale registration by FSL software [Oxford Centre for Functional Magnetic Resonance Imaging of Brain (FMRIB) Software Library, Oxford, UK].

### DTI Processing

DTIStudio 3.0.2 software (CMRM, Johns Hopkins University, Baltimore, MD, USA) was used for the reconstruction of the whole CST. Fiber tracking was performed based on fiber assignment by the continuous tracking algorithm with a multiple region of interest (ROI) approach. The ROIs were drawn in the CST area of the posterior limb of internal capsule (PLIC) and the primary motor cortex, and this included the hand motor area with fractional anisotropy (FA) color maps and the B0 image. The fiber tracts passing through both ROIs were selected as the CSTs. Fiber tracking was started at the center of a seed voxel with a FA >0.2 and ended at a voxel with a fiber assignment of <0.2 and a tract turning-angle of >70°. The apparent diffusion coefficient (ADC), FA, relative anisotropy (RA), and volume ratio (VR) were measured based on the extracted fiber tracts in the patient with CBPS and the normal subjects (3 cases).

## Results

**Fig 1:**
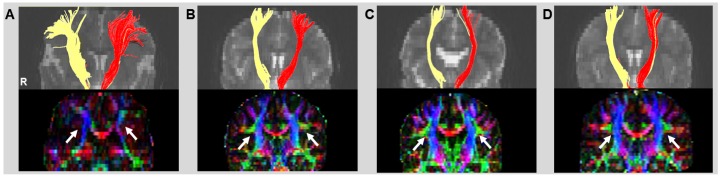
Tractography and color fractional anisotropy (FA) map of the whole corticospinal tract (CST) in a patient with congenital bilateral perisylvian syndrome (CBPS) (A, C) and a normal subject (B, D). The tracts are color coded for laterality: yellow (right) and red (left). The white arrows indicate the presence of the superior longitudinal fasciculus (SLF).

Fig 1 shows the tractography of the whole CST and the pathways in the patient with CBPS (A) and three normal subjects (B, C and D). The yellow and red fibers indicate the right and left direction, respectively, of the CST. Fig 1(A) shows that the fiber tracts were generally located in the area of the hand motor cortex in the patient with CPBS regardless of the crossing fiber effects compared to the normal subjects (B, C, and D). The numbers of fibers on both directions are 1,052 for CBPS patient, 436, 395 and 487 for three normal subjects, respectively. The normal appearance of the SLF (white arrows) in the normal subject (B, C and D) is shown with green triangular shapes lateral to the blue descending fibers of the CST. The colored map of the patient with CBPS (A) demonstrates the absence of the SLF, and the white arrows point to the estimated location where they should be found.

**Fig 2:**
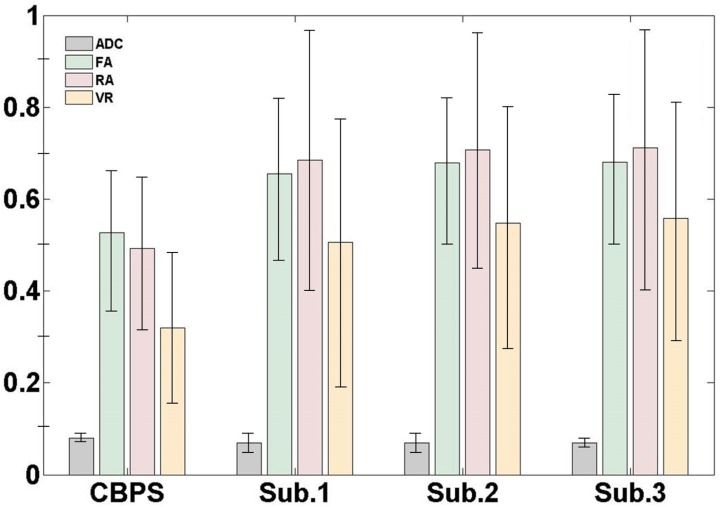
The measured quantitative values of the apparent diffusion coefficient (ADC), FA, relative anisotropy (RA), and the volume ratio (VR) for one patient with CBPS and three normal subjects. The values represent the mean (± standard deviation).

Fig 2 shows the means and standard deviations of the ADC, FA, RA, and VR values. The ADC values between the normal subjects and the patient with CBPS were not significantly different. The FA, RA, and VR values in the normal subjects were similar to each other and relatively higher compared to the patient with CBPS. The percentage differences in the FA, RA, and VR values among the subjects were about 20%, 30%, and 40%, respectively.

## Discussion

In this study, we examined the CST pathway in a patient with CBPS and compared it with the CST in normal subjects in order to visualize the CST pathway that is normally not completely reconstructed due to crossing fibers. Our results clearly show the impact of the crossing fiber for the hand motor fibers of the CST pathway with deterministic tracking algorithms in diffusion tensor tractography.

Moreover, we present for the first time the whole CST in a patient with CBPS who did not have the bilateral SLF and evaluated the quantitative values for the whole extracted CST. The quantitative values definitely indicated that there was a difference between them.

Unlike the deterministic fiber tracking, the probabilistic fiber tracking algorithms are less affected by crossing fibers. These are alternative methods to avoid the crossing fiber effects to reconstruct fibers, and comparison studies between two fiber tracking methods using these methods will also be concerned in our future works.

This finding, which was analyzed in the context of the associated clinical findings, may help us to understand the clinical presentation of CBPS. In addition, the correlation of this finding with clinical analyses may contribute to the understanding of the effects of crossing fibers.

## References

[1] Park C-h, Kou N, Boudrias M-H, Playford ED, Ward NS (2013) Assessing a standardised approach to measuring corticospinal integrity after stroke with DTI. NeuroImage: clinical 2: 521–533. 24179804 10.1016/j.nicl.2013.04.002 PMC3777681

[2] Vargas P, Gaudron M, Valabrègue R, Bertasi E, Humbert F, et al (2013) Assessment of corticospinal tract (CST) damage in acute stroke patients: Comparison of tract-specific analysis versus segmentation of a CST template. Journal of Magnetic Resonance Imaging 37: 836–845. 10.1002/jmri.23870 23086724

[3] Yeo SS, Choi BY, Chang CH, Kim SH, Jung Y-J, et al (2012) Evidence of corticospinal tract injury at midbrain in patients with subarachnoid hemorrhage. Stroke 43: 2239–2241. 10.1161/STROKEAHA.112.661116 22700530

[4] Bernal B, Rey G, Dunoyer C, Shanbhag H, Altman N (2010) Agenesis of the arcuate fasciculi in congenital bilateral perisylvian syndrome: a diffusion tensor imaging and tractography study. Archives of neurology 67: 501–505. 10.1001/archneurol.2010.59 20385920

[5] Saporta AS, Kumar A, Govindan RM, Sundaram SK, Chugani HT (2011) Arcuate fasciculus and speech in congenital bilateral perisylvian syndrome. Pediatric neurology 44: 270–274. 10.1016/j.pediatrneurol.2010.11.006 21397168

[6] Gropman A, Barkovich A, Vezina L, Conry J, Dubovsky E, et al (1997) Pediatric congenital bilateral perisylvian syndrome: clinical and MRI features in 12 patients. Neuropediatrics 28: 198–203. 9309709 10.1055/s-2007-973700

